# The complete mitochondrial genome of Amur ide (*Leuciscus waleckii waleckii*)

**DOI:** 10.1080/23802359.2019.1679679

**Published:** 2019-10-21

**Authors:** Huanqing Zhang, Dajie Xu, Lupeng Shi, Huashan Dou, Weilai Sha

**Affiliations:** a College of Life Science, QuFu Normal University, QuFu, P. R. China;; b Hulunbuir Academy of Inland Lakes in Northern Cold & Arid Areas, Hulunbuir, P. R. China

**Keywords:** *Leuciscus waleckii waleckii*, mitochondrial genome, phylogenetic analysis

## Abstract

In this study, the complete mitochondrial genome of *Leuciscus waleckii waleckii* was sequenced and got a whole length of 16605 bp. This genome was contain 2 rRNA, 22 tRNA, 13 protein-coding genes, 1 control region (D-loop) and 1 replication origin. And the nucleotide composition of this mitochondrial genome is 27.72% for A, 26.28% for T, 27.23% for C and 18.77% for G. To clarify the phylogenetic relationship of the *Leuciscus waleckii waleckii*, we concluded the phylogenetic tree using 12 PCGs (except *ND6*) of mitochondrial genome in *Leuciscus waleckii waleckii* and 16 other cyprinid fish by Bayesian inference (BI) methods and maximum-likelihood (ML). And the result show that *Leuciscus waleckii waleckii* was close to other *Leuciscus* species, especially *Leuciscus baicalensis*.

The *Leuciscus waleckii waleckii* is classified under order Cypriniformes, family Cyprinidae and genus *Leuciscus* and inhabit the Hulun lake in Inner Mongolia in China, Amur River basinin Russia, Mongolia and Korea (Xu et al. 2013). In this study, the tissue sample of *Leuciscus waleckii waleckii* was collected through field survey in Hulun Lake National Nature Reserve, Inner Mongolia, China, and the geo-spatial coordinates are 48°22′19.14″N latitude and 117°31′41.56″E longitude. The sample died a natural death. The specimen was frozen in ultra-low temperature freezer and stored in the Animal Specimen Museum of Qufu Normal University, Qufu, Shandong, China. And the accession number is QFA20180007. Then we used DNeasy Blood & Tissue kit (QIAGEN, Germantown, MD) to extract its DNA. And we held the proper permits to conduct all these sampling procedures and experimental manipulations. The mitochondrial genome got an accession number MN105127 in GenBank after we annotated it and submit it to NCBI.

The complete mitochondrial genome of *Leuciscus waleckii waleckii* is a closed circle of 16605 bp in length, and there are 37 genes in it. It contains 2 ribosomal RNA genes (rRNA), 22 transfer RNA genes (tRNA) and 13 protein-coding genes (PCGs), which is similar to other vertebrate (Zhao et al. 2016; Yang et al. 2019). There are 8 tRNA (*tRNA^Gln^*, *tRNA^Ala^*, *tRNA^Asn^*, *tRNA^Cys^*, *tRNA^Tyr^*, *tRNA^Ser^*, *tRNA^Glu^* and *tRNA^Pro^*) and only 1 PCGs (*ND6*) are located in the light strand (L-strand), and other genes are located in the heavy strand (H-strand). The nucleotide composition of *Leuciscus waleckii waleckii* is 27.72% for A, 26.28% for T, 27.23% for C and 18.77% for G. It is similar to other cyprinid fish that the nucleotide composition of A + T (54%) is higher than C + G (46%). And the gene structure, content and arrangement are also similar to other cyprinid fish reported in early research (Zhang et al. 2009).

While *Misgurnus anguillicaudatus* (MG938590) was chosen as an out-group we concluded the phylogenetic tree using 12 PCGs (except ND6) (Saitoh et al. 2000) of mitochondrial genome in Leuciscus waleckii waleckii and 16 other cyprinid fish with Bayesian inference (BI) methods and maximum-likelihood (ML) by using MrBayes v3.2.6 (Huelsenbeck and Ronquist 2001) and RaxML (Stamatakis 2014), respectively. And we choose the best model (GTR + I + G) based on the AIC criterion by testing in MrModeltest version 3.7. Then we got the same topology though we calculated it by different methods (BI and ML), and they gave a strong support for all these nodes. Finally, we concluded that *Leuciscus waleckii waleckii* was close to other Leuciscus species, especially *Leuciscus baicalensis* (Figure 1) through result of phylogenetic tree shown. We expect the data of this study to provide a useful for further research and phylogenetic relationship of Cyprinidae.

**Figure 1.**
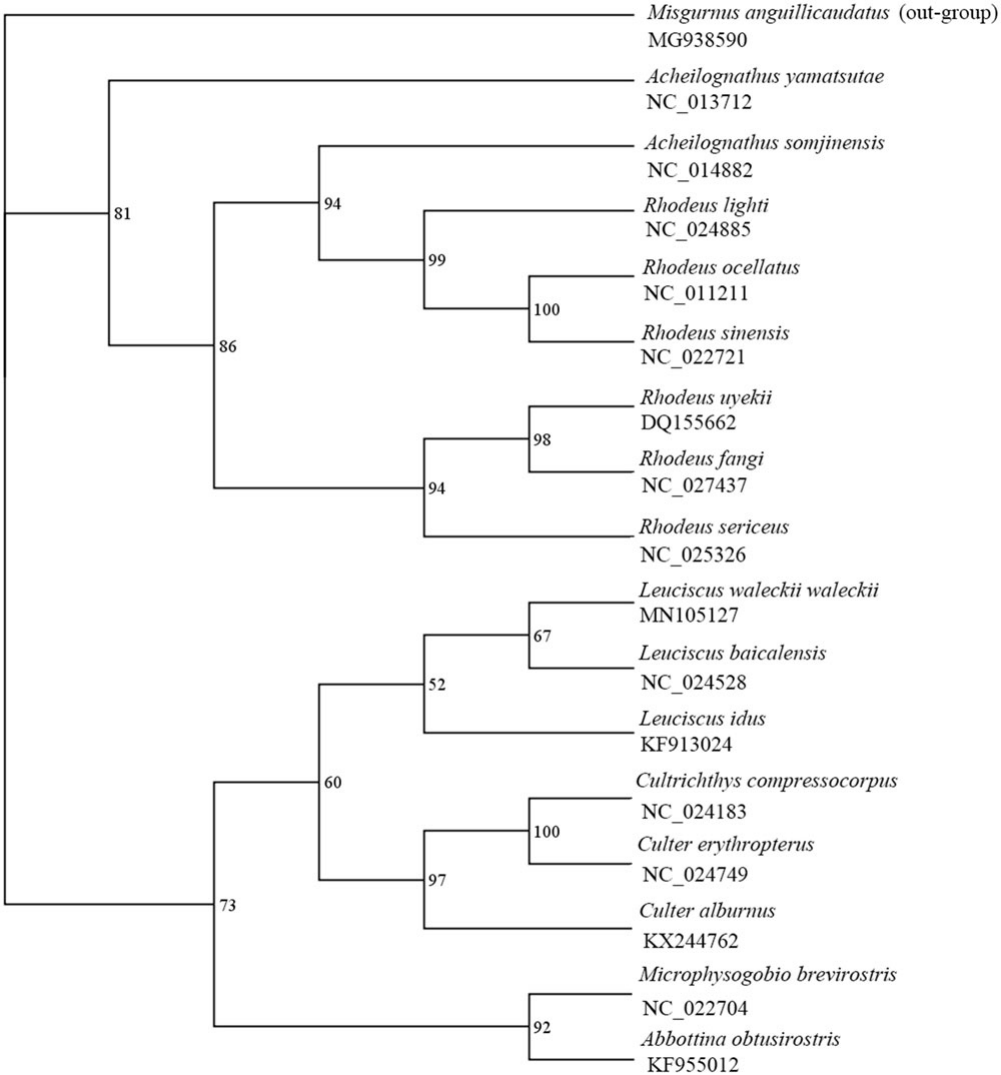
Bayesian phylogenetic inference (BI) trees of 17 species based on 12 protein-coding genes except for *ND6* and the posterior probabilities are shown on the nodes. The accession numbers of 17 species are MG938590 (*Misgurnus anguillicaudatus*), NC_013712 (*Acheilognathus yamatsutae*), NC_014882 (*Acheilognathus somjinensis*), NC_024885 (*Rhodeus lighti*), NC_011211 (*Rhodeus ocellatus*), NC_022721 (*Rhodeus sinensis*), DQ155662 (*Rhodeus uyekii*), NC_027437 (*Rhodeus fangi*), NC_025326 (*Rhodeus sericeus*), MN105127 (*Leuciscus waleckii waleckii*), NC_024528 (*Leuciscus baicalensis*), KF913024 (*Leuciscus idus*), NC_024183 (*Cultrichthys compressocorpus*), NC_024749 (*Culter erythropterus*), KX244762 (*Culter alburnus*), NC_022704 (*Microphysogobio brevirostris*), KF955012 (*Abbottina obtusirostris*).

## References

[CIT0001] Huelsenbeck JP, Ronquist F 2001 MRBAYES: Bayesian inference of phylogenetic trees. Bioinformatics, 17:754–755. 10.1093/bioinformatics/17.8.754 11524383

[CIT0002] Saitoh K, Hayashizaki K, Yokoyama Y, Asahida T, Toyohara H, Yamashita Y 2000 Complete nucleotide sequence of Japanese flounder (*Paralichthys olivaceus*) mitochondrial genome: structural properties and cue for resolving teleostean relationship. Journal of Heredity, 91:271. 10.1093/jhered/91.4.271 10912672

[CIT0003] Stamatakis A 2014 RAxML version 8: a tool for phylogenetic analysis and post-analysis of large phylogenies. Bioinformatics, 30:1312–1313. 10.1093/bioinformatics/btu033 PMC3998144 24451623

[CIT0004] Xu J, Ji P, Wang B, Zhao L, Wang J, Zhao Z, Zhang Y, Li J, Xu P, Sun X 2013 Transcriptome sequencing and analysis of wild amur Ide (*Leuciscus waleckii*) inhabiting an extreme Alkaline-Saline lake reveals insights into stress adaptation. PLoS One, 8:e59703. 10.1371/journal.pone.0059703 PMC3613414 23573207

[CIT0005] Yang X, Ao W, Xu D, Feng S, Zhang H 2019 The complete mitochondrial genome of Daurian pika (Ochotona dauurica)[J]. Mitochondrial DNA Part B, 4:519–520.

[CIT0006] Zhang X, Yue B, Jiang W, Song Z 2009 The complete mitochondrial genome of rock carp Procypris rabaudi (Cypriniformes: Cyprinidae) and phylogenetic implications. Molecular Biology Reports, 36:981. 10.1007/s11033-008-9271-y 18496768

[CIT0007] Zhao C, Zhang H, Liu G, Yang X, Zhang J 2016 The complete mitochondrial genome of the Tibetan fox (*Vulpes ferrilata*) and implications for the phylogeny of Canidae. Comptes Rendus Biologies, 339:68–77. 10.1016/j.crvi.2015.11.005 26868757

